# Multidisciplinary Studies of Folk Medicine “Five Thieves’ Oil” (Olejek Pięciu Złodziei) Components

**DOI:** 10.3390/molecules26102931

**Published:** 2021-05-14

**Authors:** Przemysław Siejak, Wojciech Smułek, Farahnaz Fathordobady, Anna Grygier, Hanna Maria Baranowska, Magdalena Rudzińska, Łukasz Masewicz, Małgorzata Jarzębska, Piotr T. Nowakowski, Aleksandra Makiej, Pardis Kazemian, Paweł Drobnik, Barbara Stachowiak, Maciej Jarzębski, Anubhav Pratap-Singh

**Affiliations:** 1 Department of Physics and Biophysics, Faculty of Food Science and Nutrition, Poznań University of Life Sciences, Wojska Polskiego 38/42, 60-637 Poznań, Poland; przemyslaw.siejak@up.poznan.pl (P.S.); hanna.baranowska@up.poznan.pl (H.M.B.); lukasz.masewicz@up.poznan.pl (Ł.M.); 2 Institute of Chemical Technology and Engineering, Poznan University of Technology, Berdychowo 4, 60-637 Poznań, Poland; wojciech.smulek@put.poznan.pl (W.S.); makiej.aleksandra@gmail.com (A.M.); 3 Food, Nutrition and Health, University of British Columbia, 2205, East Mall, Vancouver, BC V6T 1Z4, Canada; 4 Department of Technology of Plant Origin Food, Faculty of Food Science and Nutrition, Poznan University of Life Sciences, ul. Wojska Polskiego 31, 60-624 Poznań, Poland; anna.grygier@up.poznan.pl (A.G.); magdalena.rudzinska@up.poznan.pl (M.R.); barbara.stachowiak@up.poznan.pl (B.S.); 5 Poznan University of Life Sciences, 60-624 Poznań, Poland; malgorzata.jarzebska@o2.pl; 6 Institute of Pedagogy, Rzeszów University, Jałowego 24, 35-010 Rzeszów, Poland; ptnowakowski@ur.edu.pl; 7 Department of Biology, Faculty of Sciences, University of British Columbia, 1103-6270 University Blvd, Biological Sciences Building, Vancouver, BC V6T 1Z4, Canada; p.kazemian@alumni.ubc.ca; 8 Department of Orthopedics and Traumatology, Poznan University of Medical Sciences, 28 Czerwca 1956 135/147, 61-545 Poznań, Poland; drobnikp@gmail.com

**Keywords:** antibacterial activity, essential oils, *Pseudomonas*, spectrometry, terpenes, health education

## Abstract

To meet the growing interest in natural antibacterial agents, we evaluated the physicochemical and biological properties of the folk medicine known as “five thieves’ oil” (Polish name: olejek pięciu złodziei). Five thieves’ oil consists of a mixture of five oils: rosemary, lemon, clove, eucalyptus, and cinnamon. In this study, we performed gas chromatography, FTIR, and UV–vis spectroscopic analysis, as well as L-a-b color tests, contact angle determination, and surface tension determination. To verify its antibacterial activity, the metabolic activity and changes in cell membrane permeability of bacteria of the genus *Pseudomonas* were studied. As a result, it was found that among the constituent oils, the oils of clove and cinnamon were the least volatile and, at the same time, had the strongest antibacterial activity. However, a mix of all the oils also showed comparable activity, which was even more pronounced for the oils after 4 weeks of aging. This effect can be linked to the high content of terpene derivatives such as eugenol and cinnamaldehyde, which can cause changes in bacterial membrane permeability, affecting cell activity and survival. This study is the first to characterize the constituents of the popular folk medicine five thieves’ oil, confirming and explaining its strong antibacterial activity, thus constituting a significant contribution to contemporary health education.

## 1. Introduction

Since the earliest civilizations, plant extracts along with their derivatives, such as essential oils (EOs), have been widely known and used in folk medicine. First, their usage spread across the East and the Middle East, and then they also found common application in North Africa and Europe [1]. Five thieves’ oil, the origins of which are traced back to the 15th century, when the black plague ravaged Europe, is an example of recipes that can be considered an expression of traditional medicine. Anecdotes [2] narrate that four French thieves had found a quick way to wealth in robbing the sick and the dead of any valuables that could be exchanged for food and money. They knew the healing properties of various spices, and they created an essential oil blend. When applied to the hands, ears, temples, feet, and around the mouth, the oil protected them from infection.

Thieves’ oil (hereafter named oil mix) is actually a combination of several essential oils (EOs), mostly from cinnamon, clove, eucalyptus, lemon, and rosemary. Generally, EOs are made of concentrated natural products with a strong odor that are generated within aromatic plants as secondary metabolites and stored in secretory cells, cavities, canals, glandular trichomes, or epidermic cells. These oils occur as variable mixtures of primarily terpenoids, particularly monoterpenes (C10) and sesquiterpenes (C15), but diterpenes (C20) may be present as well. A broad diversity of other molecules is also found in their composition, such as acids, alcohols, aldehydes, aliphatic hydrocarbons, acyclic esters, or lactones. Rather rarely, nitrogen- and sulfur-containing compounds are present. In many traditional cultures, EOs constitute a significant part of the traditional pharmacopoeia. With the rise in consumer demand for organic products, the incorporation of EOs is becoming increasingly popular as a source of natural antioxidants, antiseptic, fragrances, preservatives, analgesics, sedatives, anti-inflammatories, spasmolytics, and local anesthetics [3,4,5,6,7,8,9,10].

The Swiss reformer of medicine Paracelsus von Hohenheim officially introduced the term “essential oil” in the 16th century. In nature, EOs mainly perform the function of protecting plants. What is more, they also attract the attention of some insects to promote the dispersion of pollens and seeds or to repel other undesirable insects [11].

EOs include a broad range of secondary metabolites that contribute to inhibiting or slowing the growth of bacteria, molds, and yeasts [12,13,14]. EOs along with their constituents have a wide spectrum of targets, especially the membrane and cytoplasm, and in some instances, they completely change the morphology of the cells. The effectiveness of essential oils has been proved to vary with their types and with the kind of target bacteria (Gram-positive and Gram-negative bacteria). For instance, the oils of *M. armillaris* and *M. acuminata* have been proven to affect the Gram-negative bacteria *E. coli* [15]. Another example constitutes sandalwood and vetiver oils, which indicate higher inhibitory activity against Gram-positive bacteria. On the other hand, they are unsuccessful in inhibiting Gram-negative bacterial strains [16,17]. The essential oils of cinnamon, clove, rosemary, pimento, thyme, and oregano have demonstrated a significant antibacterial activity against *Salmonella typhi*, *Staphylococcus aureus*, and *Pseudomonas aeruginosa* [18]. The antimicrobial effect of these oils was found to be associated with the dominant presence of components such as carvacrol, thymol, cinnamic aldehyde, eugenol, and *p*-cymene [19].

Therefore, exploitation of essential oils seems to be an effective possible solution to combat various infectious diseases and to control epidemic multidrug-resistant pathogenic microorganisms [20]. So far, applications of EOs include aromatherapy, as well as treatment of several diseases such as diabetes, Alzheimer’s, cancers, and cardiovascular diseases [21]. During the spread of SARS-CoV-2, many compositions of essential oils were explored as adjunct treatments [22]. 

Traditional medicine (also known as folk medicine) encompasses culture-specific ethnomedical beliefs and practices as well as traditional medical approaches accepted in broader cultural contexts [23]. Nowadays, this raises questions about the efficacy, and even safety, of various specifics, which pose a challenge for health education [24,25]. Nevertheless, these days, various analytical techniques can be applied for the validation of folk medicine component formulations. In this paper, we present the physicochemical studies of the five oils whose composition is traditionally named five thieves’ oil (focusing on the commercially available composition), which may propose health benefits. Our research can be considered a milestone for further development of the innovative carriers for EOs, which evaporate quite fast. Furthermore, we wanted to highlight that some “old specifics” based on traditional medicine might possibly be applied for biomedical and cosmetic applications, but still, there is a need to evaluate and verify their properties. The main aim of the study was to characterize the oil mix components from the nutritional, antibacterial, and consumers’ point of view. For this reason, we made spectroscopic and chromatographic analyses in this study, with non-standard L*a*b* color and contact angle measurements, which should be considered a first milestone for further analysis. We completed our investigation with bacterial activity tests.

## 2. Results and Discussion

### 2.1. Volatile Compounds in Fresh and Stored Essential Oils

The oil mix (“five thieves,” or “pięciu złodziei”), including rosemary, lemon, clove, eucalyptus, and cinnamon oils, was tested for its volatile profile. The percentage of the 35 volatile compounds identified in five thieves’ oil is shown in Table 1. The main volatile compounds included limonene (31%), eugenol (17%), and α-pinene (16%). The threshold values for these compounds are 10 ppb, 6 ppb, and 6 ppb, respectively. Besides, other volatiles were identified such as linalool (8%), linalyl anthranilate (7%), β-caryophyllene (4%), γ-terpine (3%), α-terpineol acetate (3%), and β-pinene (2%). Four oils were characterized by a high percentage of α-pinene: rosemary (30%), lemon (12%), eucalyptus (21%), and cinnamon (9%). No α-pinene was found in clove essential oil. The high percentage of limonene in the oil mix was related to limonene content in the lemon essential oil (12%). Limonene was also found in clove essential oil at a level of 1%. The high content of eugenol in the oil mix was associated with significant content (60%) of eugenol in clove essential oil. Eugenol was also present in cinnamon essential oil. The percentages of dominant compounds in oils were consistent with previous research studies [26,27,28,29]. Some compounds found at a lower content (menthone, methyl salicylate, linalyl anthranilate, carvacrol, and α-terpineol acetate) in the oil mix were not recorded in any of the five essential oils. This may be due to the overlapping of the peaks in the chromatogram. Based on the results presented in Table 1, it can also be seen that some compounds present in individual oils were not detected in five thieves’ essential oil.

After 4 weeks of storage at 25 °C, the analyzed oils evaporated at different rates. Among them, only clove essential oil did not evaporate. The level of evaporation of the oil mixture (82.8% of the oil remained) was similar to the value observed for cinnamon oil (77.8% left mass). Rosemary oil had a slightly higher volatility than other oils, and after 4 weeks, 50.0% of the initial mass remained. The highest loss of essential oils was observed for eucalyptus and lemon oils. Oils from eucalyptus and lemon (left mass 2.0% and 21.1, respectively) were found to be most volatile. The amounts remaining after storage were not sufficient for the determination of volatile compounds in these two essential oils. Moreover, these results correspond with the microscopic studies of the oils and the wetting property examinations (discussed in Section 2.4 and Section 2.5, respectively).

The contents (%) of individual volatile compounds in the oil mix—rosemary, clove, and cinnamon oils—after one month are presented in Table 1. The oil mix showed significant changes in eugenol, linalool, and linalyl acetate contents, which reached around 26%, 19%, and 19%, respectively. Storage of rosemary and cinnamon also led to the increase in linalool content by 6% and 18%, respectively. Similar results were obtained for lemon balm (*Melissa officinalis* L.) and basil (*Ocimum basilicum* L.) essential oils, wherein storage at room temperature increased the percentage of linalool [30,31]. During storage time, an increase in the percentage of eugenol was observed in the oil mix and cinnamon essential oil. At the same time, camphor showed a higher level in rosemary essential oil. The same results with an increased percentage of eugenol and camphor were reported for lemon balm during storage life [31]. 

In rosemary essential oil, some volatile compounds encountered some changes during storage. For example, the percentage of α-pinene was reduced from 30% to 0.13% and the same reduction was observed for camphene and β-pinene. However, the percentages of linalool, α-terpineol, β-caryophyllene, bornyl acetate, copaene, isoterpinolene, 1-hexen-3-ol, and isobornyl alcohol increased in rosemary essential oil during storage. Among volatile components, the eucalyptol content remained constant in this essential oil. The amount of volatile compounds in cinnamon oil also presented some changes during storage at 25 °C. The percentage of α-pinene, eucalyptol, and o-cymene decreased. Yet, an increase was observed in the level of linalool, α-terpineol, cinnamaldehyde, and β-caryophyllene. Clove oil was found to be the most stable essential oil in the mixture. The percentages of individual volatile compounds in clove oil did not show significant changes after one month of storage. According to previous research [31,32], the decrease in the percentage of some compounds can be related to evaporation, oxidation, and other undesirable reactions. This is confirmed in our research, wherein for the essential oil that did not evaporate during storage, the percentage of individual ingredients remained constant.

### 2.2. Low-Field NMR Studies

All investigated oils were characterized by one spin–lattice T1 and one spin–spin T2 relaxation times (Table 2). These results suggest that only protons from fatty acids were present in the system. All systems are free of water. The spin–lattice R1 and spin–spin R2 relaxation rates were calculated as an inverse of the relaxation times T1 and T2, respectively. These results are shown in Table 2. All values of both relaxation times are similar. This is the characteristic of proton-containing liquid systems that transfer energy to adjacent spins in a slightly shorter time than to the environment. For free water containing no impurities, these values, measured at the same frequency and temperature, are *R*_1_ = 0.36 s^−1^ and *R*_2_ = 40 s^−1^, respectively. The comparable values of both relaxation times allow the conclusion that only fatty acid protons are present in the system.

One of the analyzed oils is an oil mixture. Therefore, we analyzed how the values of the relaxation times of the mixture system reflected the relaxation of protons in individual oils. For this purpose, it was assumed that the relaxation rate of protons in the mixture is the sum of the relaxation rates of individual components. It is expressed by the dependence (Equation (1))

(1) $$R_{1,2}=\sum_{i=1}^{n}p_{i}R_{\left(1,2\right)i}$$

where *p_i_* is the fraction of protons belonging to the individual components of the mixture, and *R* is the relaxation rate (1 and 2, respectively). 

The results of the calculations show that in the mixture the content of the oils rosemary, eucalyptus, and cinnamon is slightly higher than 20%. At the same time, in the mixture, the content of clove and lemon oils is less than 20%.

### 2.3. Spectroscopic Analysis

**Figure 1:**
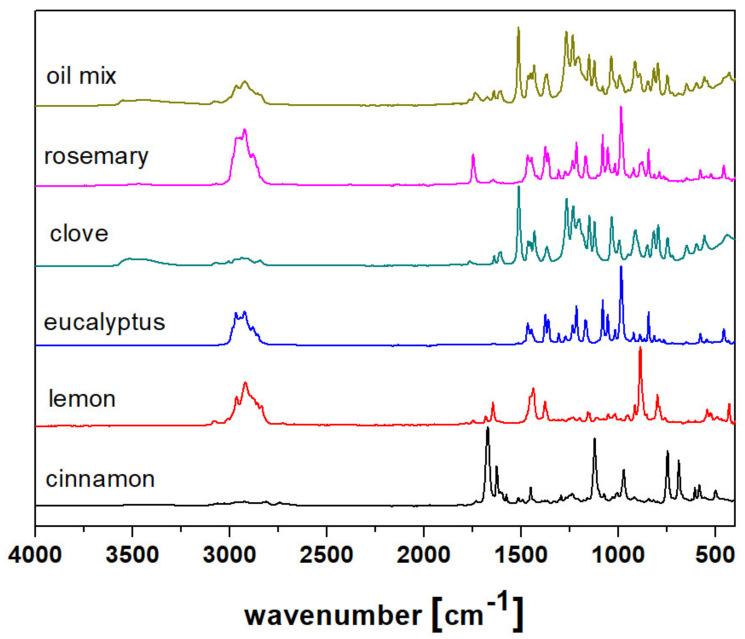
FTIR spectra of the examined oils.

Further investigations focused on infrared spectra analysis (Figure 1). Among the most dominant bands, the signal between 2800 and 3000 cm^−1^ is very noticeable and represents the stretching vibrations of the C–H bonds of aliphatic chains. The low intensity of cinnamon and clove oils is caused by the domination of aromatic compounds in these oils. The signal of the C=O bond is significant mainly for cinnamon, but also for lemon and rosemary oils (at 1700 cm^−1^), confirming the presence of aldehydes in them. Moreover, the bands from the deformation of C–H bonds, from the aromatic ring, and C–O bonds were observed at lower wavenumbers; however, as complex compositions of volatile compounds were analyzed, it was difficult to assign several bands for specific groups and compounds. Furthermore, the relatively low intensity of signals above 3000 cm^−1^ is usually a result of hydroxylic groups, the low abundance of alcohols, and the rarity of hydrogen bonds in oils. Nevertheless, the comparison of their infrared spectra suggests that in five thieves’ oil, the dominant component is clove oil, because their spectra are quite similar. The characteristic signals of other oils (mentioned bands at 2800 cm^−1^ or 1700 cm^−1^) were observed in the spectrum of five thieves’ oil as well. The spectra of eucalyptus and rosemary oils were investigated by Pollard et al. [33]. They also observed the main bond characteristic for terpenes dominated in the oils, such as those representing strong C–H and relatively weaker C=O stretching vibrations. The same signals were noticed in the cinnamon oil spectrum [34]. However, they noticed bands from O–H vibrations, which was not observed in our study. Similarly, Nagaraju et al. [35] observed the presence of the hydroxylated group in samples of clove oil. It indicated directly that strong differences between the samples from various sources may occur.

**Figure 2:**
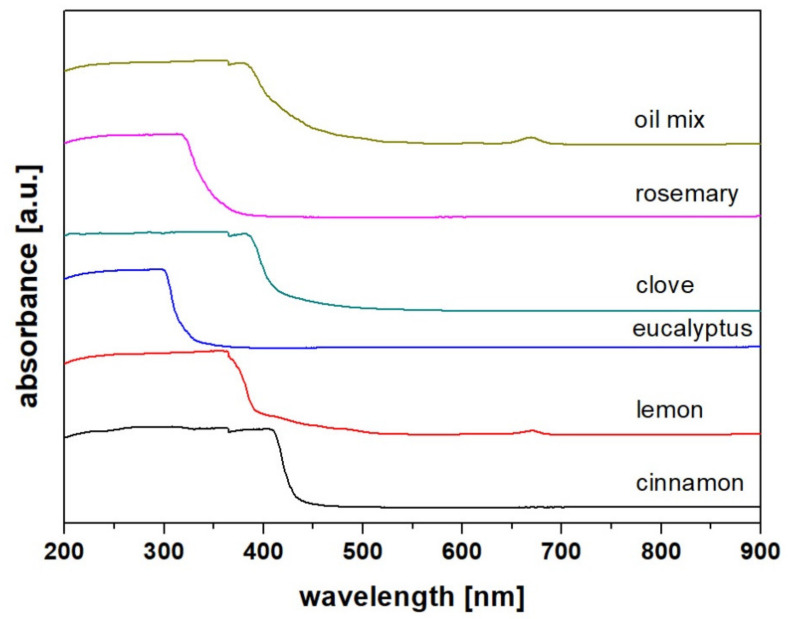
UV–vis spectra of the examined oils.

The UV–vis spectra (Figure 2) of each individual oil are typical for volatile, edible, and/or fossil oils. The spectra of most oils regardless of their origin show high absorbance in the UV region, with constant absorbance ending sharply at 320–450 nm (depending on the oil composition) and dropping sharply to 0 [36,37]. For some oils, especially edible or green plants, an additional peak at 650–700 nm assigned to chlorophylls appears. In the case of the assessed oils, only lemon and the oil mix showed this peek. It proved that lemon (or something similar) is present in the oil mix in a considerable amount. Moreover, the high-absorption region of the oil mix covers all corresponding high-absorption regions of the individual oils, and the threshold is not as sharp as it is for each oil, separately. This leads to the conclusion that the mixture consists of all five oils.

**Figure 3:**
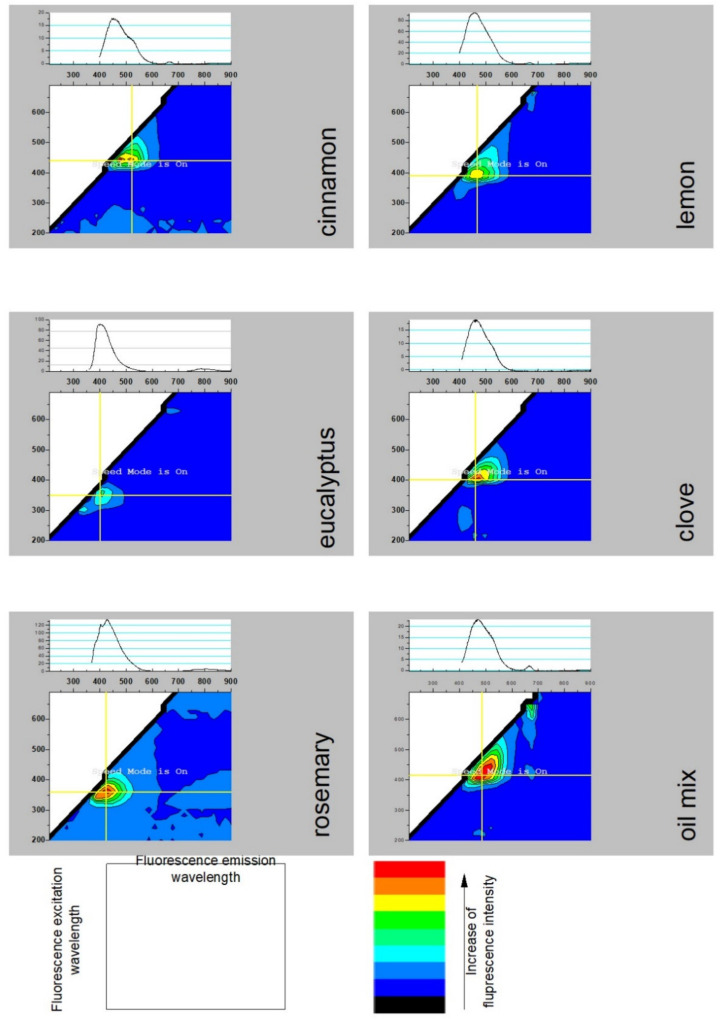
Fluorescence patterns and representative fluorescence emission spectra of the examined oils.

Figure 3 presents the fluorescence patterns and the most representative fluorescence spectra (with the highest fluorescence intensity) of the examined samples. The abscissa axis represents the emission wavelength corresponding to the selected excitation wavelength, presented as the ordinate axis. The fluorescence spectra were collected while increasing the excitation by 10 nm. 

Fluorescence patterns reveal that each of the examined oils constitute a complex pattern of several fluorescent agents. Moreover, many complex interactions between them take place, especially when energy is absorbed. The high dependence of the emission spectra on excitation wavelengths presenting as different colors in the patterns supports this conclusion. More visible individual areas with different emission spectra represent more complexity of the composition and interactions. The most complex composition is the oil mix, which consists of less complex compounds. A comparison of the patterns and the representative fluorescence emission spectra (regarding both peak position and fluorescence intensity) leads to the conclusion that the steady-state fluorescence behavior of the mix is most similar to that of clove, with the noticeable addition of lemon and cinnamon oil—the maxima of the fluorescence bands of all three are at wavelengths of 420–450 nm, and fluorescence intensities are similar. The presence of noticeable amounts of cinnamon and lemon compounds is indicated by the fluorescence area at 650–670 nm [38]. The amount of highly fluorescent compounds in the investigated samples was lower, or the interactions occurring in the oil mixture led to the quenching of fluorescence. 

### 2.4. Microscopic and Color Studies

**Figure 4:**
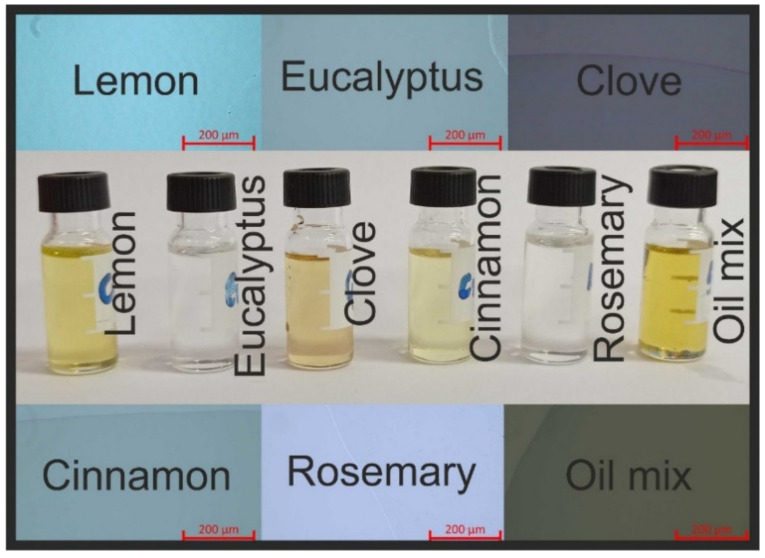
Camera image (in the middle) and microscopic images (around) of the EOs and the oil mix.

Figure 4 presents the differences between the appearances of the delivered oils. Visual observation of the oils’ homogeneity was validated using an inverted microscope. Typically, one or two droplets were put on the microscopic glass, which was cleaned with acetone. All investigated oils and the oil mix were homogeneous. As can be seen in the microscopic images in Figure 4, different light intensities were applied for phase boundary determination. During imaging, we observed that some of the oils, such as lemon, eucalyptus, rosemary, and oil mix, spread on the glass very quickly. This observation corresponds with our observations regarding evaporation speed. Based on our results, we recommend using closed cells (cuvettes) for EO studies using a microscope. The additional cover should slow down the evaporation process and protect the microscopic glass from full wetting. Moreover, it is recommended to store the essential oils in dark glass vials, which protect them from light [39]. One of the solutions for increasing the EOS stability might be their incorporation into the biopolymeric matrix. Siahbalaei et al. [40] proposed the gelatin–pectin composite as a matrix for functional materials, which could be applied for food and cosmetic products and as a carrier for EOs in biomedical applications. Due to the antibacterial properties of thyme oil, Kamrudi et al. [41] proposed nanocarriers based on polyamidoamine dendritic polymer (PAMAM). The polymeric matrix enhanced the control over releasing active compounds (here EOs) during the time period. Incorporation of EOs and EO composition, such as the “five thieves” studied here, into polymeric, biopolymeric, or emulsion systems might positively extend the delayed release effect. What is more, till now there have been limited data on the visible or confocal microscopic imaging of EOs. Typically presented results have focused on the incorporation of EOs in different structures, and most of the images were taken using scanning or transmission electron microscopes [42].

**Figure 5:**
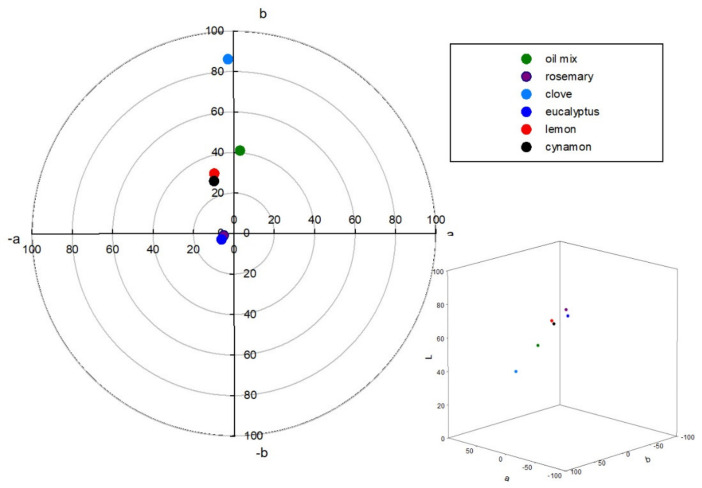
L*a*b* color space diagram of the oils.

**Figure 6:**
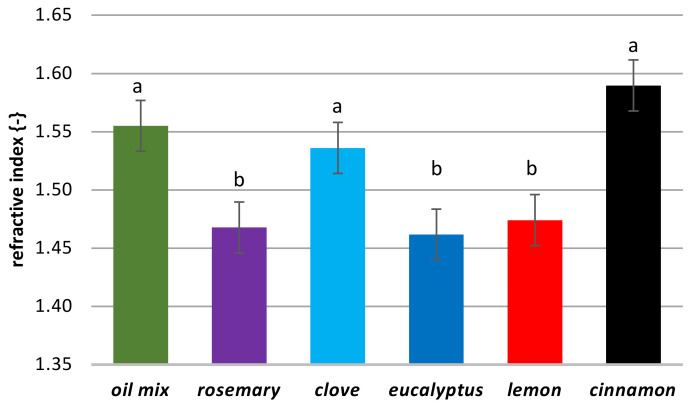
Refractive index of the oils. The different small letters indicate groups of the results that differ statistically significantly.

L*a*b* test results (see Figure 5) showed that lemon and cinnamon oils with positive values for b* and negative values for a* had a yellow-green color. Clove oil, with the highest positive value for b*, had more yellow intensity. Color components of eucalyptus and rosemary oils are in the center of the color space diagram, showing that these oils are colorless. The most intensive color was observed for the oil mix, with a green tendency (positive a*). Color changes help to evaluate the quality of oil as well as EOs. What is more, color changes of EOs might be applied as an identifier of food product quality, such as of lettuce [43]. In Figure 6, differences in the refractive index (RI) are seen. For transparent oils, such as eucalyptus and rosemary, the RI was 1.462 and 1.468, respectively. A very similar RI was determined for lemon oil (1.474). The highest RI was 1.590, for cinnamon, which was a value similar to the previously obtained 1.50 ± 0.02 and presented in [44]. An almost average RI was determined for the oil mix: 1.555 measured (arithmetic average from all oils: 1.506).

### 2.5. Surface Properties

**Figure 7:**
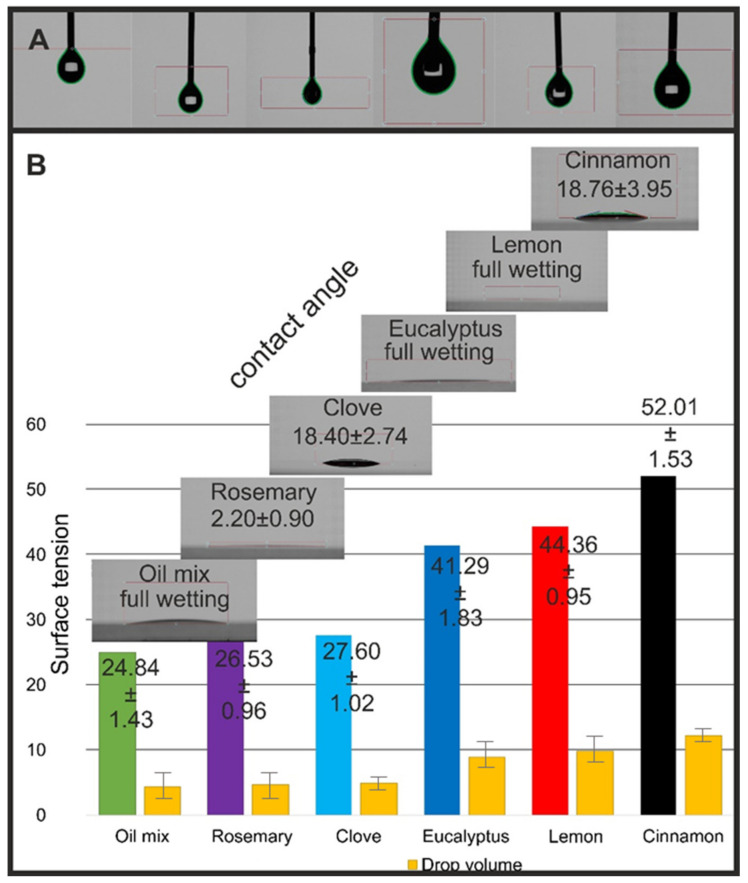
(**A**) EO droplets during surface tension measurements. (**B**) EO contact angle, surface tension, and droplet volumes values.

Optical and microscopic studies have shown that the investigated EOs can wet glass very quickly. Till now, there have not been enough data about the surface properties of EOs. Figure 7 shows the surface properties of the investigated EOs. Contact angle measurements validated a previous microscopic observation that eucalyptus, lemon, and oil mix show full wetting of the glass surface (the software could not recognize the droplet edges). Rosemary oil has similar properties, but in this case, we recorded an average contact angle of 2.20°. Opposite effects were observed for clove and cinnamon, whose contact angles were around 18°. The results indicate that EOs and the oil mix have hydrophilic properties with respect to glass. 

It must be highlighted that in this study, the results of surface activity were obtained from the samples as delivered without any additional solvents. Figure 7A presents EO droplets during pendant droplet analysis (PDA). The highest surface tension was recorded for cinnamon oil, and it corresponds with the largest registered droplet volumes and contact angle value. The surface tension values of eucalyptus and lemon oils were 41.29 and 44.36, respectively. Surprisingly, the lowest values of surface tension were recorded for the oil mix (24.84). Significant differences in surface tensions were observed between the samples with smaller and bigger droplet volumes. For further surface activity evaluation, more detailed studies are recommended. However, the collected results correspond with the general knowledge about the hydrophobic properties of essential oils [45].

The surface properties of EOs can be improved by adding suitable surfactants. Haba et al. [46] demonstrated that rhamnolipids promote surface properties and the dispersion of EOs by reducing the surface tension, thus enhancing their functionality and antimicrobial activity against *Candida albicans* and methicillin-resistant *Staphylococcus aureus* (MRSA).

### 2.6. Antimicrobial Activity

**Figure 8:**
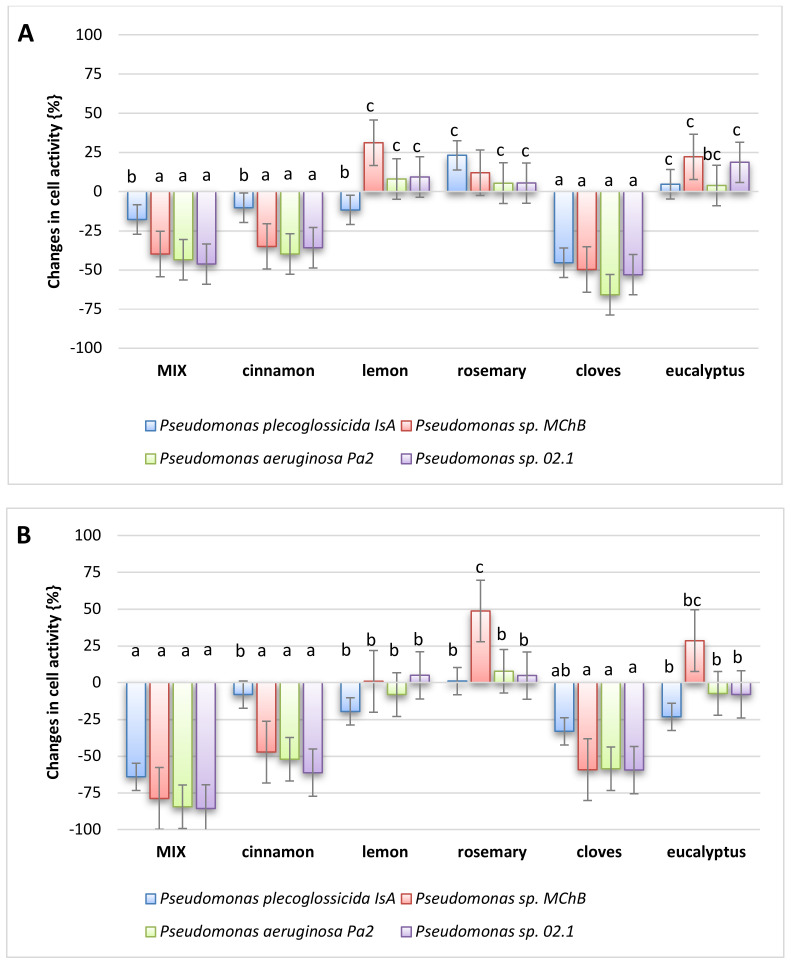
Changes in bacteria cell metabolic activity in (**A**) fresh oils, and (**B**) oils after 4 weeks. The different small letters indicate groups of the results that differ statistically significantly.

The final stage of the conducted research was to evaluate the effect of essential oils on spoilage microorganisms, from the *Pseudomonas* genus, which are ubiquitous and may cause some wound infections (e.g., *P. aeruginosa*). The results obtained were interesting as they showed an ambiguous bactericidal effect of the oils (Figure 8). When examining fresh oils, it should be noted that the strongest toxic effect against bacterial strains of the genus *Pseudomonas* was shown by clove oil (over 50% decrease of cell metabolic activity). A slightly weaker antibacterial effect was shown by cinnamon oil and five thieves’ oil. Interestingly, the components of the other three oils had a non-toxic effect, but even, to some extent, stimulated the metabolic activity of the bacterial cells. The results of the toxic effects of the residual oils after 4 weeks of weathering are intriguing. The antibacterial effect of five thieves’ oil is more intense, and eucalyptus and lemon oils also began to show a slightly toxic effect against bacteria (but not exceeding 20%). However, these changes can be explained by the fact that weathering increased the relative proportion of less volatile components in the oils. As our study indicates, it is these that are responsible for the antibacterial effects of essential oils. This also corresponds with the observation that the most antibacterial oils, clove and cinnamon, are also the oils that showed the least weight loss after 4 weeks. 

**Figure 9:**
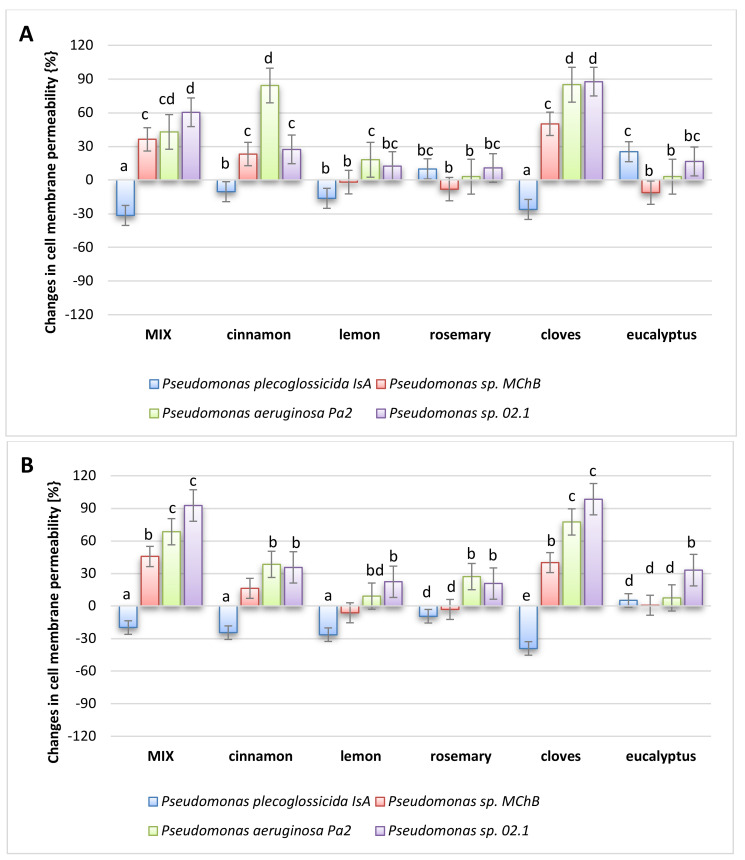
Changes in bacteria cell membrane permeability in (**A**) fresh oils, (**B**) oils after 4 weeks. The different small letters indicate groups of the results that differ statistically significantly.

Due to the hydrophobic nature of the oils, it was assumed that their antibacterial effect might be related to their interaction with the phospholipid bacterial membrane. Therefore, additional studies were conducted to investigate the effect of the tested oils on the permeability of bacterial membranes of selected environmental strains (Figure 9). It should be emphasized that the results obtained are not as conclusive as measurements of cell metabolic activity. In this case, the differences are rather determined by the type of bacterial strain and not so strongly by the type of oil. However, taking into account the average values, it should be pointed out that the oils with the strongest antibacterial activity caused the strongest increase in cell membrane permeability. This confirms the a priori accepted hypothesis, but at the same time, it can be stated that more precise studies are necessary to clarify the real mechanism of the antibacterial action of oils.

The antibacterial effects of five thieves’ oil have not yet been analyzed. However, the bioactive properties of the essential oils forming the tested composition have already been studied independently. Clove oil was the subject of the research presented by Alexa et al. [47], who found it to be against the strain *Pseudomonas aeruginosa* (ATCC 27853). It inhibited the growth of microorganisms already at a concentration of 2 µg/100 mL.

Some of the studies also included research on the interaction of oils, such as cinnamon and clove. In their publication, Bahurmiz et al. [48] did not observe any synergistic effect indicative of increased antibacterial activity. However, Purkait et al. [49] observed it, on the contrary, against *P. aeruginosa* as well. 

For rosemary oil, the minimal inhibitory concentration against *P. aeruginosa* was over 40 µg/mL [50]. Elcocks et al. [51] performed extensive analyses of the bactericidal activity of a range of oils against selected bacterial strains. Cinnamon and clove oils were effective against *P. aeruginosa*, but not rosemary and lemon oils, which are quite similar results to those presented in our study. Moreover, Kozics et al. [52] compared the effects of different oils. Eucalyptus and clove oils had similar effects against the *Pseudomonas* strain, but significantly weaker than oregano or thyme oil.

When comparing the results obtained, however, it should be kept in mind that antibacterial tests were performed using different methods and against different strains of microorganisms. Therefore, it was so important to repeat these analyses in parallel with the oil of “five thieves” under the same conditions. 

## 3. Materials and Methods

### 3.1. Materials

Rosemary, clove, eucalyptus, lemon, and cinnamon oils were purchased from Avicen-na-Oil (Wrocław, Poland). As a reference sample, the oil mix (traditionally called five thieves’ oil; in Polish, “olejek pięciu złodziei”) was purchased from Natura Receptura (Elbląg, Poland). For the tests, all oils were used as delivered.

### 3.2. Methods

#### 3.2.1. GCMS

Volatile compounds from samples were isolated using headspace solid phase microextraction (HS-SPME) using a method detailed earlier in [53] with slight modifications. Each oil (1.5 mL) was placed in different vials (vials volume: 15 mL). The oils were heated for 5 min at 40 °C. SPME fiber divinylben-zene/carboxen/polydimethylsiloxane (DVB/CAR/PDMS; Supelco, Bellefonte, PA, USA) was used for extraction. The fiber was firstly conditioned in the GC injection port at 270 °C for 4 h. To absorb volatiles, fiber was exposed in vials with samples for 5 min at 40 °C in split-less mode. Desorption was carried out at the GC injection port for 5 min. The volatiles were analyzed on a 7890A GC System with a triple-axis mass detector (Agilent Technologies, Santa Clara, CA, USA). Injector temperature was 280 °C. A capillary column SLB5MS (30 m × 0.25 mm × 0.5 µm; Merck, Darmstadt, Germany), with helium as a carrier gas at a flow rate of 0.8 mL/min, was used. The initial oven temperature was 40 °C for 3 min, which was then increased to 160 °C at 4 °C/min. Finally, the temperature was increased to 280 °C at a rate of 10 °C/min, and it was held for 5 min. Mass spectra were recorded in an electron impact mode (70 eV), and masses were scanned from 33 to 333 Da. Mass spectra of volatile components were compared with the mass spectra from MS library–NIST MS Search 2.0.

#### 3.2.2. Essential Oil Evaporation Tests

The tested essential oils were placed in vials with a capacity of 15 mL in one repetition. The open vials were placed in a laboratory oven, set at 25 °C. The samples were left in the oven for 4 weeks.

#### 3.2.3. Low-Field NMR

The samples at volume equal to 0.15 mL were put in the NMR tube and closed using Parafilm^®^. The NMR relaxation times, spin–lattice T1 and spin–spin T2, were measured using a pulse spectrometer PS15T (Ellab, Poznan, Poland) operating at 15 MHz. The T1 relaxation times were measured using inversion-recovery (IR) pulse sequence. T2 were measured by using the Carr–Purcell–Meiboom–Gill (CPMG) method [54]. These methods are typical for food and food component investigations. Calculations of the spin–lattice relaxation times were performed with the assistance of the CracSpin program [55]. The spin–spin relaxation times were calculated using a computational specific program dedicated to the spectrometer using the least-squares method. For all samples, time T1 and time T2 were obtained. These suggest that in all oils, only protons from fatty acid chains are present.

All measurements were performed at a stabilized temperature of +20 °C. The measurements were replicated three times. For each experiment, new samples from the same bottle were prepared.

The results are shown as the mean values of relaxation parameters.

#### 3.2.4. FTIR 

The FTIR spectra were obtained using a Spectrum Two FT-IR spectrometer equipped with a Universal ATR with a diamond crystal (PerkinElmer, Waltham, MA, USA). The data were collected over a spectral range of 4000–500 cm^−1^. The measurements were repeated three times for each sample.

#### 3.2.5. UV–Vis 

The UV–vis spectra were recorded for the wavelength range 200–900 nm with the use of a Shimadzu UV–VIS 1201 spectrophotometer (Kyoto, Japan). The spectra were recorded for bulk oils inserted into a quartz cuvette (HELMA) with an optical path of 1 mm. All the measurements were performed at ambient conditions.

#### 3.2.6. Fluorescence Behavior 

The fluorescence spectra of each of the samples were recorded using a Shimadzu RF 5001PC fluorometer (Kyoto, Japan) under ambient conditions. All the spectroscopic measurements were performed under ambient conditions, in a 1 cm × 1 cm quartz cuvette (HELMA). The samples were excited at UV and VIS regions, and emission was measured over the range up to 990 nm with 3 nm excitation and emission slits. A 90° (L-shaped) geometry excitation-to-emission beam was used.

#### 3.2.7. Microscopic Investigations and Camera Imaging 

The microscopic investigations were performed by using the inverted microscope ZEISS Axio Vert.A1 (Zeiss, China). The studies were performed with the objective magnifications LD A-Plan 20x/0,35 (air) and color camera Axiocam 208 (Zeiss, China). For imaging, the oils were put on microscopic glass. For the presentation, the resolution of the images was automatically adjusted as the best fit using ZEN2.5 software (Zeiss, Germany).

#### 3.2.8. L*a*b* Color Properties

For color evaluation, an NH310 portable spectrophotometer (Shenzhen ThreeNH Technology Co., Ltd., Shiyan, China) equipped with internal software was applied. Before examination, 2 mL of the sample was inserted into the transparent plastic cuvette. The measurements were carried out in a cuvette inserted into a dedicated measurement chamber. The color tests were repeated 10 times, and average values with SD were recorded.

#### 3.2.9. Refractive Index 

The refractive indexes (RIs) were determined using an analogous optical Abbe Refractometer (RL, Polskie Zakłady Optyczne S.A., Warsaw, Poland). The measurements were performed in triplicate.

#### 3.2.10. Contact Angle and Surface Tension Determination

An Ossila contact angle goniometer (Ossila Ltd., Sheffield, UK) was applied for the measurements of the contact angles and surface tensions of oil droplets. One droplet of the selected essential oil or oil mix was dropped on the microscopic glass surface for the contact angle measurements. For surface tension measurements, a glass syringe with 25 µL volume and blunt-tipped needle of 0.47 mm diameter was applied. The average values of 10 measurements were presented. For the pendant droplet measurements, the droplet density was uniformly adjusted by software for acetone (0.791 g/mL; due to high speed of evaporation, no density measurements of EOs were performed; air density 0.00128 g/mL). The measurements were performed at room temperature.

#### 3.2.11. Antibacterial Effect of Oils 

To determine the biocidal activity of the tested essential oils, two types of tests were performed using four environmental strains of the genus *Pseudomonas: Pseudomonas* sp. MChB, *Pseudomonas aeruginosa* Pa2, *Pseudomonas plecoglossicida* IsA, and *Pseudomonas* sp. 02.1. All these strains came from the microbial collection of the Department of Organic Chemistry of Poznan University of Technology. 

Cell suspension in minimal medium [56] obtained after centrifugation of cells from 24 h culture in nutrient broth at 30 °C was used for analysis. The tested oils were added to the suspension at a concentration of 0.5% (*v*/*v*). The control sample was a cell suspension without the addition of any essential oil.

The first assay included the measurement of cell metabolic activity using the MTT indicator, similar to what is described in [57]. Further analyses included a study of changes in cell membrane permeability. For this purpose, a crystal violet assay was used, following the methodology presented in [56].

### 3.3. Statistical Analysis

In all analyses, at least three independent experiments were performed, unless otherwise indicated. The mean values were accepted as final results. The statistical significance of differences between the mean values was determined by ANOVA (one-way analysis of variance) with Tukey’s range test applied as post hoc analysis. Differences at *p* < 0.05 were considered statistically significant. The calculations were performed using Statistica v13 (StatSoft, Cracow, Poland).

## 4. Conclusions

Due to high demand for new antibacterial and antiviral agents, exploration of the traditional system of medicines is necessary. Here, we focused on the surface activity and biological activity tests for the traditional formula of five thieves’ oil. The results showed the possibility of enhancing desirable properties by changing the ratio of EOs in the oil mix composition. Limonene (31%), eugenol (17%), and α-pinene were the most prominent volatiles present in the oil mix responsible for their antibacterial behavior, which is primarily attributed to lemon oil, clove essential oil, and rosemary, respectively. During one month of storage, the volatile content decreased rapidly, with limonene and α-pinene becoming non-detectable, whereas eugenol and linalool percentage increased considerably. This points to the changing nature of the oil composition with time and storage, which needs to be the focus of future studies. Furthermore, we observed the beneficial effects of the different EO mixtures. One of the crucial findings of our study was to evaluate the spectroscopic and antibacterial properties of the five thieves’ oil mix and its components. Most of the surface properties of the oil were found to be very similar to those of rosemary oil. As presented in this study, the tale of five thieves’ oil composition might be interesting for further bioactive compound development. Plant derivatives attract great attention for possible use in the fight against microbial pathogens, especially due to SARS-Cov-2. Our results show that clove and cinnamon oils possess strong antimicrobial properties. The results are one of the first milestones in developing new solutions to applying essential oils and their various compositions. Still, there is an additional need for studies on new carriers for desirable tuning of delayed or targeted release systems.

## Figures and Tables

**Table 1:** Percentage of volatile compounds in fresh and storage oil mix, with rosemary, lemon, clove, eucalyptus, and cinnamon essential oils.

Compound	Kovat’s Index	Fresh						After 1 Month			
		Oil Mix	Rosemary	Lemon	Clove	Eucalyptus	Cinnamon	Oil Mix	Rosemary	Clove	Cinnamon
1-Hexen-3-ol	851								1.11		
2-Thujene	873	0.45					4.04				
α-Pinene	939	16.46	30.06	11.66		20.65	9.34		0.13		
Camphene	953	1.41	8.66	0.5			1.2				
Benzaldehyde	961						1.44				1
β-Pinene	980	2.05	13.28	28.48		7.95		2.12	3.11		0.14
2-Carene	1001	0.65					0.48				
o-Cymene	1022						17.94		0.5		
m-Cymene	1025				0.21					0.06	3.72
Sylvestrene	1027			28.69							
Limonene	1031	30.91		11.6	1.05					0.44	
Eucaliptol	1033		23.66			60.98	10.79		23.7	0.18	3.67
γ-Terpine	1062	3.43	2.12	11.01		8.46	0.8	3.07	1.74	0.05	
α-Terpinolene	1088		1.36	2.15		0.89					
Isoterpinolene	1090							7.64	2.07		
Linalool	1098	7.53	1.24	0.54			11.25	18.69	5.76		17.71
1-Ethyl-4-Isopropyl-Cyclohexyl 2-Hydroperfluorobutanoate	1120							1.21			
Camphor	1143	0.94	10.06					1.98	26.99		
Borneol	1165		2.02						11.23		
α-Terpineol	1189	0.35	0.36	0.59		1.08	1.08	2.54	5.92		2.13
Linalyl Acetate	1248							18.57			
Cinnamaldehyde	1266	1.95					30.98				53.24
Bornyl Acetate	1285		2.33						5.79		
Carvacrol	1298	0.69						1.34			
β-Citral	1316			2.09							
α-Terpineol Acetate	1340	3.05						1.76			
α-Citral	1341	0.33		2.47				0.56			
Eugenol	1356	16.69			60.4		2	25.69		60.49	3.99
Copaene	1376		0.42		0.72		1.07		2.19	0.1	1.94
β-Caryophyllene	1404	4.28	3.85	0.22	27.18		6.66	9.95	8.54	28.82	12.47
α-Caryophyllene	1418	0.59	0.59		6.68		0.92	1.71	1.21	7.39	
Isoeugenol	1447	0.35			2.56			0.39		1.42	
Benzalmalonic Dialdehyde	1454							1.42			
Acetyleugenol	1524	0.62			1.2			0.88		1.04	
Linalyl Anthranilate	2157	7.26						0.48			

**Table 2:** The values of spin–lattice and spin–spin relaxation rates in the investigated oils (*R*—relaxation rate; *p_i_*—fraction of protons).

Sample	*R*_1_ (s^−1^)	*R*_2_ (s^−1^)	*p_i_*
Oil mix	1.585 ± 0.015	1.612 ± 0.012	–
Rosemary	1.215 ± 0.012	1.221 ± 0.031	0.26 ± 0.02
Clove	2.383 ± 0.008	2.403 ± 0.027	0.13 ± 0.02
eucalyptus	1.274 ± 0.009	1.241 ± 0.019	0.25 ± 0.03
Lemon	1.705 ± 0.013	1.724 ± 0.021	0.18 ± 0.03
Cinnamon	1.283 ± 0.004	1.332 ± 0.019	0.24 ± 0.02

Mean values ± SD.

## Data Availability

All collected data were presented in this study.

## Sample Availability

Samples are commercially available.
